# Alternative polyadenylation drives oncogenic gene expression in pancreatic ductal adenocarcinoma

**DOI:** 10.1101/gr.257550.119

**Published:** 2020-03

**Authors:** Swati Venkat, Arwen A. Tisdale, Johann R. Schwarz, Abdulrahman A. Alahmari, H. Carlo Maurer, Kenneth P. Olive, Kevin H. Eng, Michael E. Feigin

**Affiliations:** 1 Department of Pharmacology and Therapeutics, Roswell Park Comprehensive Cancer Center, Buffalo, New York 14263, USA;; 2 Klinikum rechts der Isar, II. Medizinische Klinik, Technische Universität München, 81675 Munich, Germany;; 3 Herbert Irving Comprehensive Cancer Center, Department of Medicine, Division of Digestive and Liver Diseases, Department of Pathology and Cell Biology, Columbia University Medical Center, New York, New York 10032, USA;; 4 Department of Cancer Genetics and Genomics, Roswell Park Comprehensive Cancer Center, Buffalo, New York 14263, USA;; 5 Department of Biostatistics and Bioinformatics, Roswell Park Comprehensive Cancer Center, Buffalo, New York 14263, USA

## Abstract

Alternative polyadenylation (APA) is a gene regulatory process that dictates mRNA 3′-UTR length, resulting in changes in mRNA stability and localization. APA is frequently disrupted in cancer and promotes tumorigenesis through altered expression of oncogenes and tumor suppressors. Pan-cancer analyses have revealed common APA events across the tumor landscape; however, little is known about tumor type–specific alterations that may uncover novel events and vulnerabilities. Here, we integrate RNA-sequencing data from the Genotype-Tissue Expression (GTEx) project and The Cancer Genome Atlas (TCGA) to comprehensively analyze APA events in 148 pancreatic ductal adenocarcinomas (PDACs). We report widespread, recurrent, and functionally relevant 3′-UTR alterations associated with gene expression changes of known and newly identified PDAC growth-promoting genes and experimentally validate the effects of these APA events on protein expression. We find enrichment for APA events in genes associated with known PDAC pathways, loss of tumor-suppressive miRNA binding sites, and increased heterogeneity in 3′-UTR forms of metabolic genes. Survival analyses reveal a subset of 3′-UTR alterations that independently characterize a poor prognostic cohort among PDAC patients. Finally, we identify and validate the casein kinase CSNK1A1 (also known as CK1alpha or CK1a) as an APA-regulated therapeutic target in PDAC. Knockdown or pharmacological inhibition of CSNK1A1 attenuates PDAC cell proliferation and clonogenic growth. Our single-cancer analysis reveals APA as an underappreciated driver of protumorigenic gene expression in PDAC via the loss of miRNA regulation.

Pancreatic ductal adenocarcinoma (PDAC) is a lethal cancer with a 5-yr survival rate of 9% (Siegel et al. 2017). Extensive sequencing studies have uncovered recurrently mutated genes (*KRAS*, *TP53*, *SMAD4*, *CDKN2A*) and dysregulated pathways (axon guidance, cell adhesion, small GTPase signaling, protein metabolism) driving disease initiation and progression (Jones et al. 2008; Waddell et al. 2015; The Cancer Genome Atlas Research Network 2017). Gene expression profiles from hundreds of patient samples have allowed the identification of several PDAC subtypes, with implications for treatment response and patient outcome (Collisson et al. 2011; Moffitt et al. 2015; Bailey et al. 2016; Lomberk et al. 2018; Tiriac et al. 2018; Maurer et al. 2019). Gene expression can be dysregulated in cancer through a variety of mechanisms, including genomic amplification/deletion, epigenetic modification, and noncoding mutations in promoters/enhancers (Khurana et al. 2013; Weinhold et al. 2014; Jones et al. 2015; D'Antonio et al. 2017; Rheinbay et al. 2017). For example, recurrent noncoding mutations in PDAC are enriched in promoters of cancer-associated genes and pathways (Feigin et al. 2017). However, our understanding of the mechanisms driving dysregulated gene expression in cancer remains incomplete. Determining the regulatory mechanisms driving dysregulated gene expression is critical to understanding disease pathogenesis. One such regulatory mechanism that has recently gained recognition as a critical driver of gene expression is alternative polyadenylation (APA).

APA is a post-transcriptional process that generates distinct mRNA isoforms of the same gene as a mechanism to modulate gene expression. This includes transcripts that have identical coding sequences but vary only in the length of their 3′ untranslated region (UTR) (Elkon et al. 2013; Erson-Bensan and Can 2016; Gruber and Zavolan 2019). Changes in 3′-UTR length can modulate mRNA stability, function, or subcellular localization through disruption of miRNA or RNA-binding protein regulation (Elkon et al. 2013; Mayr 2016; Tian and Manley 2017). APA is driven by a large complex of polyadenylation factors that recognize a series of highly conserved sequences within the 3′ UTR on the newly synthesized pre-mRNA before cleavage and addition of the poly(A) tail (Proudfoot 2011; Elkon et al. 2013; Shi and Manley 2015). Because most transcripts contain multiple polyadenylation sites (PAS), the choice of where to cleave is a critical determinant of 3′-UTR length. In humans, a majority of genes (51%–79%) express alternative 3′ UTRs, demonstrating the widespread nature of this process (Mayr 2019). Indeed, APA has roles in muscle stem cell function, cell proliferation, chromatin signaling, pluripotent cell fate, cellular senescence, and other physiological processes (Sandberg et al. 2008; Boutet et al. 2012; Lackford et al. 2014; Brumbaugh et al. 2018; Chen et al. 2018a). Recently, dysregulation of APA has gained recognition as a driver of tumorigenesis (Sandberg et al. 2008; Mayr and Bartel 2009; Masamha et al. 2014; Miles et al. 2016; Chen et al. 2018b). APA factor expression is altered in a variety of cancer types and promotes tumorigenesis by regulating the expression of oncogenes (via loss of miRNA regulation) and tumor suppressors (via disruption of competing-endogenous RNA cross talk) (Masamha et al. 2014; Li et al. 2015; Chen et al. 2018b; Mitra et al. 2018; Park et al. 2018). The relevance of APA in cancer was established with the discovery of a systemic increase in the usage of a proximal PAS leading to consistently shortened 3′ UTRs of oncogenes such as insulin like growth factor 2 mRNA binding protein 1 (*IGF2BP1*), Rac family small GTPase 1 (*RAC1*), and cyclin D2 (*CCND2*) (Mayr and Bartel 2009; Chen et al. 2018b). Functional studies of the genes composing the APA machinery have highlighted their relevance to tumor growth; for example, in glioblastoma, overexpression of the APA factor *NUDT21* (a repressor of proximal 3′-UTR PAS usage) reduces tumor cell proliferation and inhibits tumor growth in vivo (Masamha et al. 2014). Subsequently, a number of pan-cancer analyses have used standard RNA-sequencing (RNA-seq) data to identify 3′-UTR shortening and lengthening events across cancer types (Xia et al. 2014; Le Pera et al. 2015; Grassi et al. 2016; Feng et al. 2017; Ye et al. 2018). Although these analyses have uncovered recurrent APA events across multiple tumor types, they also detected tumor type–specific events (Xue et al. 2018). Additionally, differential 3′-UTR processing has been shown to drive tissue-specific gene expression (Lianoglou et al. 2013). However, there has been no in-depth single-cancer analysis with a sufficiently large patient cohort to unravel disease-specific APA alterations. Furthermore, none of the pan-cancer studies have included PDAC owing to a lack of matched normal controls and therefore, the landscape of APA in PDAC remains completely uncharacterized.

To determine the relevance of APA in PDAC, we performed a comprehensive analysis of the changes in PAS usage using RNA-seq data from 148 PDAC tumors from The Cancer Genome Atlas Pancreatic Adenocarcinoma (TCGA-PAAD) study and 184 normal pancreata from the Genotype-Tissue Expression (GTEx) project (The Cancer Genome Atlas Research Network et al. 2013; The GTEx Consortium 2015). We performed a systems level analysis to identify trends in APA, impacts on gene expression, and effects of miRNA regulation. Our in-depth analysis reveals APA as a recurrent, widespread mechanism underlying oncogenic gene expression changes through loss of tumor-suppressive miRNA regulation in pancreatic cancer.

## Results

To analyze differences in APA profiles between tumor and normal samples, we selected 148 patients out of the total 178 PDAC patients with aligned RNA-seq data from the TCGA-PAAD study. We excluded 30 patients in the cohort that did not have histologically observable PDAC tumors (The Cancer Genome Atlas Research Network 2017). Due to the paucity of RNA-seq data from matched normal tissues within the TCGA-PAAD study, we procured raw RNA-seq reads from 184 normal pancreata from the GTEx project. The library preparation and sequencing platform were identical for the TCGA-PAAD study and GTEx pancreata data (The GTEx Consortium 2015; The Cancer Genome Atlas Research Network 2017), thereby minimizing potential batch effects. Several previous studies have successfully compared TCGA and GTEx gene expression data, noting minimal batch effects when processed in an identical manner (Kosti et al. 2016; Aran et al. 2017; Zeng et al. 2019). Therefore, these data sets were processed identically and analyzed for differences in APA in our downstream analyses (Supplemental Fig. S1). To allow a rigorous comparison between GTEx normal pancreas and TCGA-PAAD tumor samples, we aligned raw reads from the GTEx RNA-seq data per the TCGA pipeline. We processed the tumor and normal aligned files to generate coverage files that were used to identify 3′-UTR differences. We assessed the extent of differential batch effects by comparing the variation in expression of housekeeping genes between the two data sets (Eisenberg and Levanon 2003). We computed the median expression (log_2_[normalized counts]) of housekeeping genes from our coverage data and found a high correlation between the tumor and normal data sets (Pearson *R* = 0.91, *P* < 2 × 10^−16^) (Supplemental Fig. S2A), suggesting that the two data sets are comparable. The coverage data were used as an input for the Dynamic Analysis of Alternative Polyadenylation from RNA-seq (DaPars) algorithm (Xia et al. 2014). DaPars is a regression-based algorithm that performs de novo identification of APA events between two conditions using standard RNA-seq data (Masamha et al. 2014; Xia et al. 2014; Chen et al. 2018b). DaPars generates a Percentage Distal Usage Index (PDUI) score for a given gene for every sample. The PDUI score quantifies the relative poly(A) site usage for that gene in the sample by computing the abundances of 3′-UTR long and short forms. Genes favoring distal PAS usage (long 3′ UTRs) have PDUI scores near 1, whereas genes favoring proximal PAS usage (short 3′ UTRs) have PDUI scores near 0. The final output was a PDUI matrix containing 2573 unique genes as rows and tumor/normal sample in each column (total 148 tumor + 184 normal = 332 columns). To compare 3′-UTR changes for a given gene between tumor and normal samples, the PDUI scores for the gene were averaged over tumor (MeanPDUI_T_) and normal (MeanPDUI_N_) samples. A change in the mean PDUI score between tumor and normal samples for each gene (ΔPDUI = MeanPDUI_T_ − MeanPDUI_N_) was calculated and used as a measure of tumor-associated 3′-UTR shortening or lengthening.

### Integrative analysis of GTEx and TCGA-PAAD RNA-seq data identifies 3′-UTR shortening events associated with PDAC

**Figure 1.**
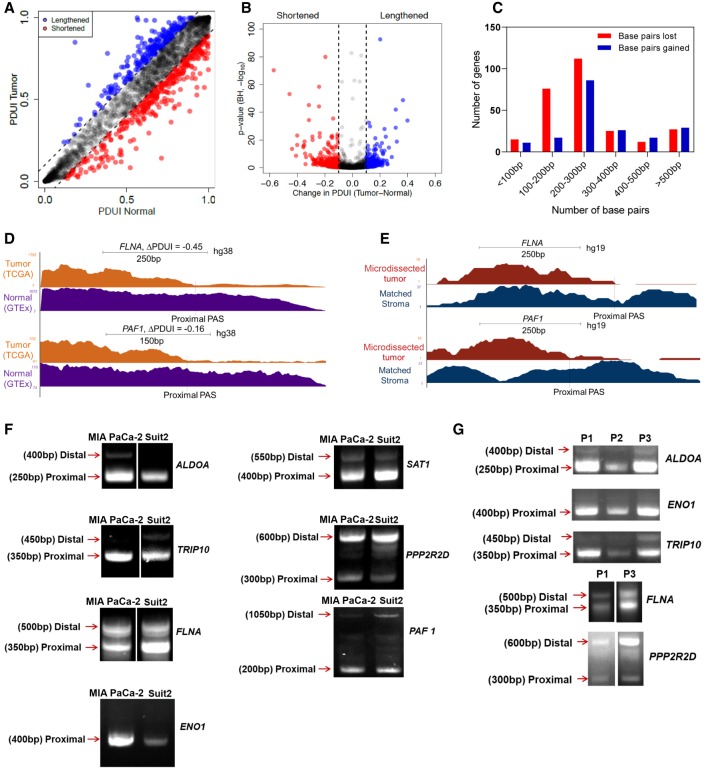
Integrative analysis of RNA-seq data identifies 3′-UTR alterations associated with PDAC. (*A*) A plot of PDUI score of each gene in human tumor and normal samples. Dashed lines represent 0.1 cutoffs. Blue dots represent 3′-UTR-lengthened genes, and red dots represent 3′-UTR-shortened genes. (*B*) A volcano plot denoting 3′-UTR-shortened (red) and -lengthened (blue) gene hits (FDR < 0.01) whose |ΔPDUI| > 0.1. (*C*) A plot showing the number of base pairs lost/gained by 3′-UTR-altered genes. (*D*) UCSC Genome Browser plot depicting the 3′-UTR RNA-seq density profile of two 3′-UTR-shortened genes (*FLNA* and *PAF1*) to highlight the coverage differences between tumor (orange) and normal (purple) patient samples. (*E*) UCSC Genome Browser plot highlighting the 3′-UTR profile differences between *FLNA* and *PAF1* in a microdissected data set in patient tumor (red) and stroma (blue). (*F*) 3′ RACE of altered PDAC-associated genes in MIA PaCa-2 and Suit2 cells (representative images, *n* = 3). Approximate length of the 3′-UTR form is denoted *beside* each band. (*G*) 3′ RACE of select genes in primary patient samples (P1, P2, P3).

To determine an appropriate ΔPDUI threshold to identify shortened/lengthened genes, we performed a permutation test (*n* = 10,000) and computed the adjusted *P*-values (*P*_adj_) for ±0.05, ±0.1, and ±0.15 thresholds. For a threshold of ±0.05, 23.8% of genes showed *P*_adj_ > 0.05, suggesting that this threshold can lead to multiple false positives. However, zero genes showed *P*_adj_ > 0.05 for the ±0.1 and ±0.15 thresholds (Supplemental Fig. S2B,C). Therefore, we chose ΔPDUI = ±0.1 as a stringent threshold to identify shortened/lengthened genes with minimum false positives/negatives. To determine the extent of APA-mediated 3′-UTR shortening and lengthening in PDAC, we compared the PDUI scores for each gene between the tumor and normal samples (Fig. 1A,B). Although the majority of genes do not undergo changes in APA, PDAC patients are characterized by a greater number of significant 3′-UTR shortening events (red dots, *n* = 266) as compared to significant lengthening events (blue dots, *n* = 186) (Fig. 1B). A higher number of 3′-UTR shortening events compared to lengthening events in PDAC is consistent with patterns observed in multiple pan-cancer analyses (Mayr and Bartel 2009; Xia et al. 2014; Xiang et al. 2018). The tumor-associated shortening and lengthening events were predominantly 100–300 bp and 200–300 bp in length, respectively (Fig. 1C). Among the genes found to have significantly shortened 3′-UTR lengths were many known PDAC growth-promoting genes, including PAF1 homolog, Paf1/RNA polymerase II complex component (*PAF1*), filamin A (*FLNA*), enolase 1 (*ENO1*), Ral guanine nucleotide dissociation stimulator (*RALGDS*), thyroid hormone receptor interactor 10 (*TRIP10*), and aldolase, fructose-bisphosphate A (*ALDOA*). *ALDOA* and *PAF1* have recently been described as oncogenes in PDAC (Dey et al. 2014; Vaz et al. 2014; Ji et al. 2016; Nimmakayala et al. 2018), whereas *ENO1*, *RALGDS*, *TRIP10*, and *FLNA* are known to mediate pancreatic cancer cell proliferation, survival and migration (Chien and White 2003; Li et al. 2009; Mihaljevic et al. 2010; Hsu et al. 2011; Zhou et al. 2011; Capello et al. 2016). We did not detect 3′-UTR alterations in recurrently mutated PDAC genes, reflecting the predominant role of APA in regulating gene expression rather than gene function. We visualized the 3′-UTR profiles of these genes between TCGA and GTEx samples to confirm 3′-UTR shortening (see *FLNA*, *PAF1* as examples) (Fig. 1D).

PDAC samples are often characterized by substantial stromal contamination (Maurer et al. 2019); therefore, we sought to determine if significant APA events were present in the stroma or the tumor epithelium. First, we determined for every significant gene hit in our analysis whether sample purity is correlated with the PDUI score. The measure of purity considered for each sample was the pathologist-reviewed tumor cellularity score (The Cancer Genome Atlas Research Network 2017). However, none of our significant gene hits showed a significant correlation (Pearson's R > 0.3, *P* < 0.05) between PDUI score and tumor purity. We then analyzed PDUI changes in a subset of 69 high purity TCGA-PAAD tumor samples (>33% tumor content) (The Cancer Genome Atlas Research Network 2017). Eighty-nine percent of gene hits from our original analysis showed up as significant hits in the high purity data set, suggesting that the majority of the detected APA changes were not attributable to stromal contamination (Supplemental Fig. S3A,B). We further addressed this concern by visualizing the 3′-UTR profile of our candidate genes in an independent data set containing RNA-seq information from 65 matched human PDAC samples with microdissected tumor epithelium and stroma (Maurer and Olive 2019; Maurer et al. 2019). As an example, Figure 1E shows the differential 3′-UTR shortening of *FLNA* and *PAF1* in patient tumor epithelium (tumor cells) as compared to the matched stroma.

We validated the presence of alternative 3′-UTR forms for several APA-regulated candidate genes by 3′ RACE (rapid amplification of 3′ ends) in two human pancreatic cancer cell lines (Suit2, MIA PaCa-2) and primary patient samples (Fig. 1F,G). These genes included the previously described PDAC growth-promoting genes, as well as the spermine/spermidine N1-acetyltransferase 1 *SAT1*, and protein phosphatase 2 regulatory subunit Bdelta (*PPP2R2D*). SAT1 modulates cell migration and resistance in multiple tumor types, whereas PPP2R2D is a component of the tumor-suppressive phosphatase PP2A (Vandenberg 2008; Seshacharyulu et al. 2013; Brett-Morris et al. 2014; Phanstiel 2018; Yu et al. 2018; Fahrmann et al. 2019). With the exception of *PPP2R2D*, which displayed significant 3′-UTR lengthening and down-regulation in tumors, all of the validated genes were significantly shortened and overexpressed in the TCGA-PAAD data set. We detected 3′-UTR short and long forms via 3′ RACE. The short 3′-UTR form for the majority of the shortened genes predominated over the long form (Fig. 1F,G). *ENO1* showed a single 3′-UTR form suggesting that this is the predominant form in cancer cells. In contrast, *PPP2R2D* showed an increased proportion of the 3′-UTR long form in PDAC cell lines and patient samples as compared to the short form, suggesting greater use of the distal PAS for this putative tumor-suppressive gene. For every candidate, we successfully identified PAS sites within its 3′-UTR sequence that matched the expected position of proximal and distal PAS in the detected 3′ RACE forms (Supplemental Fig. S3C). Therefore, a large-scale comparison of 3′-UTR alterations can identify tumor epithelium-specific changes from the TCGA and GTEx data sets, and these 3′-UTR forms can be detected in cell models and patient samples.

### 3′-UTR changes are widespread among PDAC patients and enriched in PDAC pathways

**Figure 2.**
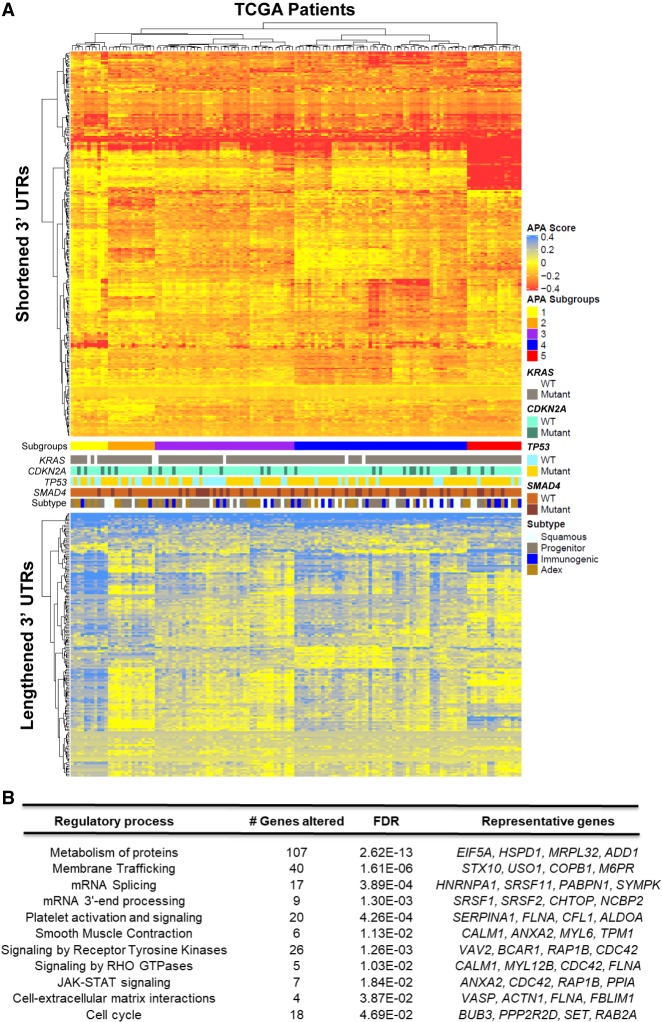
3′-UTR changes are widespread among PDAC patients and enriched in PDAC pathways. (*A*) The heatmap shows genes (rows) undergoing 3′-UTR shortening (red) or lengthening (blue) in each patient tumor (columns) compared to median score in normal pancreas for that gene. The profile of *KRAS*, *CDKN2A*, *TP53*, and *SMAD4* mutations as well as tumor subtype is shown in the context of distinct APA-derived patient subgroups. (*B*) Significantly enriched (FDR < 0.05) reactome pathways associated with 3′-UTR-altered genes.

To visualize the landscape of APA across PDAC, we clustered patients (columns) based on change in PDUI score (tumor − normal mean; ΔPDUI) for 3′-UTR-altered genes (rows) (Fig. 2A). This analysis uncovered a subset of genes (*n* = 68) that showed 3′-UTR shortening (red) in >90% of patients, highlighting the widespread nature of APA across PDAC. A smaller subset of 3′ UTRs (*n* = 26, bottom heatmap) was recurrently lengthened (blue) in the tumor cohort. Hierarchical clustering identified multiple patient subgroups characterized by 3′-UTR alterations of specific gene sets (Subgroups 1–5). Subgroup 5 was enriched in shortened 3′ UTRs and contained relatively few lengthening events. In contrast, Subgroup 1 displayed fewer 3′-UTR shortening events and was enriched in 3′-UTR lengthening. Subgroups 2–4 were characterized by shortening events in specific subsets of genes. These subgroups did not correlate with the mutational status of recurrently mutated PDAC genes (*KRAS*, *CDKN2A*, *SMAD4*, *TP53*), nor did they associate with previously described PDAC subtypes.

Pathway analysis of the significantly altered genes revealed enrichment for mRNA 3′ end processing and splicing, as well as smooth muscle contraction and platelet activation. Similar pathways have been found by pan-cancer APA analyses, concordant with the presence of recurrent APA events across multiple cancer types (Lianoglou et al. 2013; Xia et al. 2014). However, we observed further enrichments in PDAC-associated pathways, including protein metabolism, signaling by receptor tyrosine kinases, signaling by RHO GTPases, JAK-STAT signaling, and cell–extracellular matrix interactions (Fig. 2B). Therefore, APA alterations may regulate the activity of PDAC-promoting pathways.

### 3′-UTR shortening identifies a poor prognostic cohort in PDAC patients

**Figure 3.**
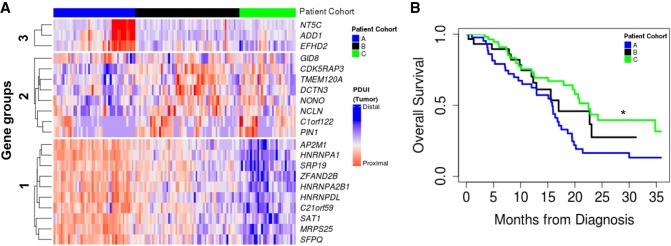
APA events identify a poor prognostic cohort in PDAC patients. (*A*) Patients were clustered based on 3′-UTR short (red) and long forms (blue) of 3′-UTR-altered genes (clustered into gene Groups 1, 2, and 3) and segregated into patient Cohort A (blue), patient Cohort B (black), and patient Cohort C (green). (*B*) Kaplan–Meier survival plot for patient Cohort A (blue), patient Cohort B (black), and patient Cohort C (green): (*) *P* < 0.05.

Next, we asked whether APA events added additional prognostic information to PDAC patient outcomes above the usual demographic and clinical factors: age, race, sex, stage, grade, and surgical outcome. We selected genes with significant 3′-UTR alterations and univariate prognostic value, defining prognostic classes based on multivariate clustering (Fig. 3A). This segregated patients into three cohorts based on their 3′-UTR patterns (long in blue; short in red). Cohort A was predominantly associated with proximal PAS usage of genes from Groups 1 and 3, whereas Cohort C was associated with distal PAS usage of the same genes. For Group 2 genes, distal PAS usage was predominant in Cohort A, but proximal PAS usage was predominant in Cohort C. Neither patient cohort correlated with any of the known PDAC tumor subtypes. Cohorts A and C displayed significant differences in overall survival, with patients in Cohort C living significantly longer than those in Cohort A (*P* = 0.003) (Fig. 3B). Therefore, patterns of APA can be used as an independent prognostic indicator in PDAC.

### Heterogeneity of proximal PAS usage of metabolic genes in PDAC patients

**Figure 4.**
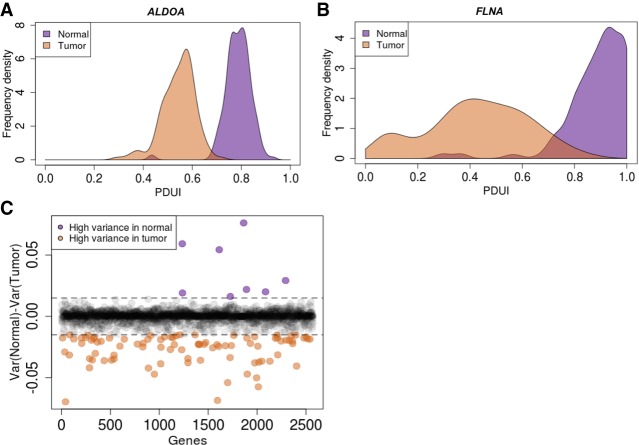
PDAC patients show substantial heterogeneity in the extent of proximal PAS usage of metabolic genes. (*A*) Example of a 3′-UTR-shortened gene (*ALDOA*) that has a tight distribution of its proximal PAS usage in normal pancreas (purple) as well as PDAC patients (orange). (*B*) A 3′-UTR-shortened gene (*FLNA*) that has a tight distribution in normal pancreas (purple); however, the extent of proximal PAS usage varies greatly across PDAC patients (orange). (*C*) Plot of variance in PDUI for all genes between tumor and normal. Purple dots represent genes with high variance in normal samples, and orange dots represent genes with high variance in tumor samples. Dashed lines represent 0.015 and −0.015 cutoffs.

Processes generating genetic and epigenetic heterogeneity can drive tumor evolution (Easwaran et al. 2014; McGranahan and Swanton 2017; Hinohara and Polyak 2019). We hypothesized that APA could represent such a process, creating a diverse set of 3′-UTR forms and allowing cancer cells to select for those that promote their survival and propagation. To examine this heterogeneity, we compared the variance of proximal PAS usage across patients in any given gene between tumor and normal samples. *ALDOA* is shown as an example gene that exhibited a tight distribution of PAS usage across normal as well as patient tumors (Fig. 4A). The left shift of the tumor sample mean score represents an increased proximal PAS usage signifying the expected shortening of the *ALDOA* 3′ UTR. However, for *FLNA*, although the normal samples had a tight distribution, PDAC patients showed greater heterogeneity in PAS usage (Fig. 4B). An analysis of heterogeneity in PAS usage for all genes revealed that although the majority of genes did not show a significant change between normal and tumor conditions, 89 genes showed greater heterogeneity in tumor (orange) samples and only eight genes showed greater heterogeneity in normal (purple) samples (Fig. 4C). This heterogeneity was not attributable to intrinsic differences between the TCGA and GTEx data sets, because none of the 215 housekeeping genes in the data set showed differences in heterogeneity in the extent of proximal PAS usage (Zhu et al. 2008; Eisenberg and Levanon 2013). The subset of 89 genes was enriched in metabolic genes, specifically amino acid transporters and purine metabolism. Increased heterogeneity of PAS usage in PDAC patients suggests a possible role of PAS usage plasticity in promoting cancer cell survival and progression.

### APA drives altered protein expression in PDAC

**Figure 5.**
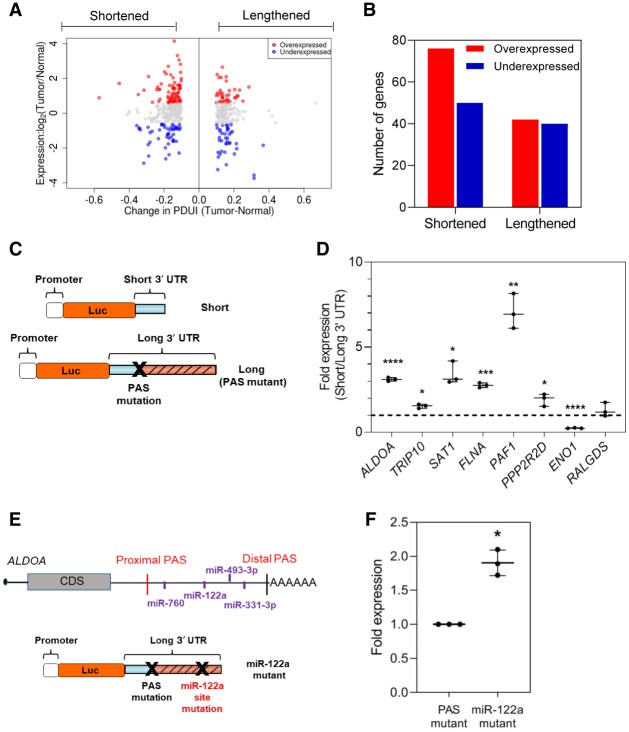
APA drives altered protein expression in PDAC. (*A*) Log fold change in gene expression is plotted against ΔPDUI for 3′-UTR-altered genes. Overexpressed genes (red dots) and underexpressed genes (blue dots) on the *left* represent 3′-UTR-shortened hits, whereas those to the *right* represent 3′-UTR-lengthened hits. (*B*) Quantification of 3′-UTR-altered genes that are overexpressed (red) or underexpressed (blue) in PDAC tumors. (*C*) Schematic illustrating the luciferase reporter constructs. (*D*) Normalized fold expression change of the luciferase reporter (short 3′ UTRs/long 3′ UTRs) for the selected list of 3′-UTR-altered genes (*n* = 3). The long 3′-UTR expression for each gene is normalized to 1. Each whisker plot denotes the median as the centerline and the minimum and maximum values as the whiskers: (*) *P* < 0.05; (**) *P* < 0.01; (***) *P* < 0.005; (****) *P* < 0.001. (*E*) Schematic showing the *ALDOA* 3′ UTR with positions of conserved miRNA sites as well as the miRNA mutant construct used. (*F*) Fold expression change of miRNA mutant construct compared to the PAS mutant in luciferase assays (*n* = 3). The PAS mutant expression is normalized to 1.

To determine whether the identified APA events drive altered gene expression in PDAC, we computed differential gene expression between normal (GTEx) and tumor (TCGA-PAAD) tissues. This allowed association studies between specific APA events and changes in gene expression. Among 3′-UTR-shortened genes, 76 were significantly up-regulated, and 50 genes were significantly down-regulated in tumors (Fig. 5A,B). Increased gene expression in the subset of 76 genes conforms to the expectation that 3′-UTR-shortened genes can escape miRNA regulation leading to increased gene expression (Lee and Dutta 2007; Mayr et al. 2007; Mayr and Bartel 2009). However, the association of 3′-UTR-shortened genes with up-regulation was not statistically significant (Fisher's exact test, *P* = 0.09). 3′-UTR-lengthened genes showed a similar number of significantly up-regulated (*n* = 42) and significantly down-regulated (*n* = 41) genes, consistent with pan-cancer analyses, and most likely reflective of positive and negative regulation by RNA-binding proteins (Matoulkova et al. 2012; Pereira et al. 2017; Chen et al. 2018a). To experimentally validate the relationship between APA and protein expression, we performed luciferase reporter assays in MIA PaCa-2 cells, comparing protein expression driven by short and long 3′ UTRs (Fig. 5C). We focused on the candidate oncogenes and tumor suppressors validated by 3′ RACE and that showed significant association between 3′-UTR changes and gene expression in tumors. These candidates included *ALDOA*, *FLNA*, *PAF1*, *TRIP10*, *ENO1*, *SAT1* (shortened and up-regulated in tumors), and *PPP2R2D* (lengthened and down-regulated in tumors). We also included *RALGDS*, which is shortened but does not show altered expression in tumors. We cloned the short and long 3′ UTRs of each gene (estimated via 3′ RACE) downstream from a *Renilla* luciferase reporter and measured luminescence as a readout of protein expression (Fig. 5C). To ensure that the long 3′-UTR form for each reporter gene remained intact (i.e., did not undergo APA-mediated shortening upon transfection into cells), we mutated their functional proximal PAS. For all genes tested except *ENO1* and *RALGDS*, the short 3′-UTR form showed significantly increased luminescence compared to the long 3′-UTR form (Fig. 5D). As predicted, the 3′-UTR short and long forms of *RALGDS* showed similar expression. In contrast to our expectations, the short form of *ENO1* showed decreased protein expression suggesting that 3′-UTR shortening is not the sole mechanism regulating protein abundance of *ENO1* in PDAC. To further show that the expression changes driven by the short and long 3′-UTR forms are governed by the sequence content and not simply a function of 3′-UTR length, we cloned the reverse complement of the long 3′-UTR segment of *PAF1* downstream from its short 3′ UTR. The proximal PAS was mutated to prevent APA-mediated shortening of this control construct (Supplemental Fig. S4A). As expected, this construct showed similar luminescence as the short 3′-UTR form, suggesting that the observed expression differences are primarily a function of sequence content within the long 3′ UTR (Supplemental Fig. S4B). These findings reinforce the observation that gene regulation is 3′-UTR sequence–dependent and that shorter 3′ UTRs do not always increase protein expression (Mayr and Bartel 2009). Overall, the above results show that APA-mediated 3′-UTR alterations can regulate the protein expression of growth-promoting genes in PDAC cells.

We next sought to determine the mechanism underlying the 3′-UTR-mediated gene regulation of the PDAC oncogene *ALDOA*. Given that miRNAs primarily destabilize their target mRNA and that *ALDOA* undergoes 3′-UTR shortening and up-regulation, we searched the *ALDOA* 3′ UTR for putative miRNA binding sites that would be lost upon PDAC-associated shortening (Fig. 5E). We identified the tumor-suppressive miRNA miR-122 within this lost region; miR-122 is expressed in PDAC cell lines (Tsai et al. 2009; Zhang et al. 2009). Mutation of the miR-122 site within the long 3′ UTR of *ALDOA* significantly restored protein expression (Fig. 5F). Therefore, altered APA can regulate oncogene expression in PDAC through modulation of available regulatory miRNA binding sites.

### APA-mediated loss of tumor-suppressive miRNA binding sites is associated with poor patient outcome

**Figure 6.**
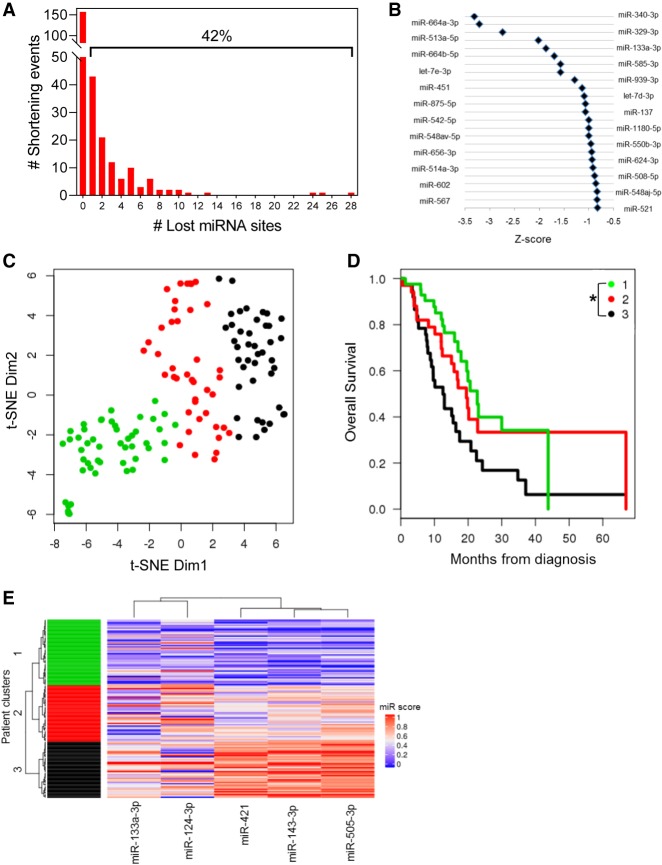
APA-mediated loss of tumor-suppressive miRNA binding sites is associated with poor patient outcome. (*A*) Number of genes that lose highly conserved miRNA binding sites because of 3′-UTR shortening. The percentage of genes that lose at least one miRNA binding site is indicated *above* the bracket. (*B*) Highly conserved miRNA families were plotted against their *Z*-score, an index of the lost binding sites in which a more negative *Z*-score indicates more significant binding site loss. (*C*) t-SNE plot depicting TCGA patient clusters in the highly conserved miRNA feature space. (*D*) Kaplan–Meier survival plot for the three patient clusters identified in *C*: (*) *P* < 0.05 for Cluster 1 to Cluster 3 comparison. (*E*) Heatmap depicting the association of miRNA binding site loss (miR score) with patient clusters.

To assess global patterns of APA-mediated miRNA binding site loss, we searched for highly conserved miRNA binding sites (conserved across human, mouse, rat, dog, and chicken) within the lost 3′ UTRs of all shortened genes. This analysis revealed that 42% of genes lost at least one highly conserved miRNA binding site (Fig. 6A), suggesting that alteration of the miRNA binding site repertoire is a common mode of APA-mediated regulation. Next, we sought to determine if any miRNA families were preferentially lost in shortened 3′ UTRs of PDAC patients. We computed an index for repression for each miRNA family as a function of the miRNA site context scores (obtained from TargetScan) and the abundance of the 3′-UTR form containing that site. This index was then compared between PDAC patients and normal controls to yield a *Z*-score. A lower *Z*-score for a miRNA family reflects preferential loss of its binding sites because of 3′-UTR shortening. We focused on the top eight miRNAs, because after the eighth miRNA, all subsequent significantly lost miRNAs had similar *Z*-scores (near −1) and all eight are expressed in pancreatic cancer cell lines (Zhang et al. 2009). Six of these top eight miRNAs have been implicated as tumor suppressors in PDAC, including miR-329 and miR-133a (Fig. 6B; Dangi-Garimella et al. 2011; Qin et al. 2014; Wang et al. 2016; Baradaran et al. 2019; Wang et al. 2019). These results suggest that APA regulates oncogenic gene expression through preferential loss of tumor-suppressive miRNA binding sites and may therefore confer a selective advantage to the cell.

Next, we determined whether loss of specific miRNA sites associated with 3′-UTR alterations is associated with patient outcome. We quantified loss of highly conserved miRNA binding sites for each patient as a function of the extent of proximal PAS usage in all genes that lost those miRNA sites (Methods). Clustering in the miRNA feature space revealed three patient groups (Fig. 6C) with significant differences in overall survival (*P* = 0.012 between Clusters 1 and 3) (Fig. 6D). We also performed the analysis of deviance test of the nested Cox regression model: clinical variables versus clinical + miRNA clusters. We found that addition of miRNA clusters significantly improved the model. In terms of magnitude of effect, the hazard ratios associated with the miRNA clusters (Cluster 1/Cluster 3 as reference) were HR = 0.59 and HR = 0.44. For context, the strongest significant clinical effect is HR = 0.52 for R0 surgical status. This suggests the miRNA-usage-based modeling is at least as strong as clinical variables. The miRNAs most significantly associated with the patient clusters included miR-133a, miR-124, miR-421, miR-143, and miR-505. Binding sites for each miRNA were preferentially lost from Cluster 1 as compared with Cluster 3, suggesting that loss of these regulatory sites correlates with poor survival of PDAC patients (Fig. 6E). Indeed, miR-133a, miR-124, and miR-143 are known tumor suppressors in PDAC, again supporting the role of APA in selective loss of tumor-suppressive miRNA binding sites (Kent et al. 2010; Hu et al. 2012; Kojima et al. 2012; Pham et al. 2013; Qin et al. 2014; Schultz et al. 2014; Wu et al. 2018).

### The APA-regulated gene *CSNK1A1* is required for proliferation and clonogenic growth of PDAC cells

**Figure 7.**
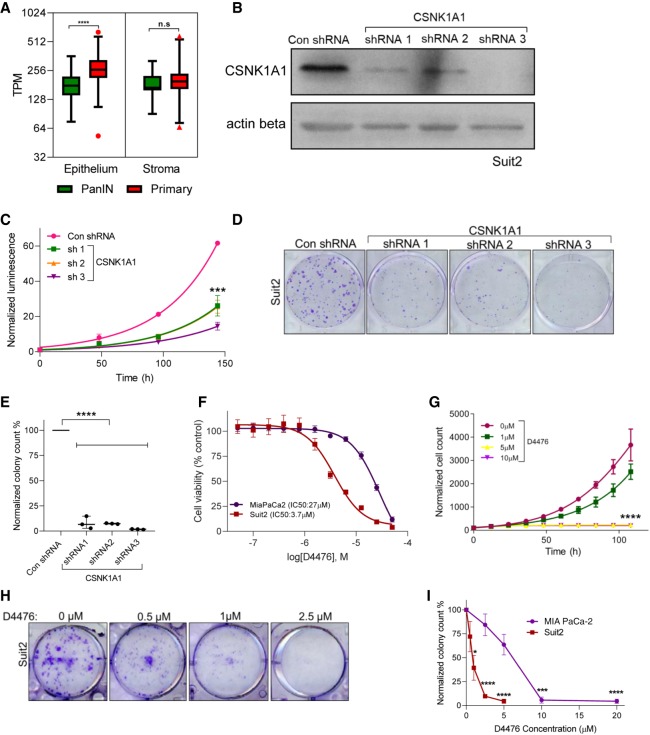
CSNK1A1 is required for cell proliferation and is a putative drug target in PDAC. (*A*) A plot showing *CSNK1A1* gene expression (in transcripts per million) in PDAC (red) as compared to PanIN lesions (green) in the epithelium and stroma from microdissected samples: (****) *P* < 0.001. (*B*) A representative blot confirming CSNK1A1 knockdown in Suit2 cells with a nontargeting control shRNA (Con shRNA) or with one of three different shRNAs targeting *CSNK1A1*: *n* = 3. ACTB (also known as actin beta) is shown as a loading control. (*C*) Cell proliferation of Suit2 control and *CSNK1A1*-knockdown cells: *n* = 3; (***) *P* < 0.005. (*D*) Clonogenic growth assay of control and *CSNK1A1*-knockdown cells: *n* = 3. (*E*) Quantification shows the number of colonies in *D*: *n* = 3; (****) *P* < 0.001. (*F*) Dose-response of MIA PaCa-2 (purple) and Suit2 (red) cell lines to the CSNK1A1 small molecule inhibitor, D4476: *n* = 3. (*G*) Cell proliferation of Suit2 cells treated with indicated doses of D4476: *n* = 3; (****) *P* < 0.001. (*H*) Clonogenic growth assay of Suit2 cells treated with indicated drug doses. (*I*) Quantification shows the number of colonies in *H*: (***) *P* < 0.005; (****) *P* < 0.001.

Our analyses revealed APA-mediated regulatory changes in genes known to promote PDAC pathogenesis. We hypothesized that our altered gene list may also contain growth-promoting genes not previously implicated in PDAC biology, and therefore new therapeutic targets. We focused on the subset of druggable genes that were significantly shortened and up-regulated in PDAC. This analysis identified *CSNK1A1*, the gene encoding the serine/threonine kinase casein kinase 1 alpha 1 (CSNK1A1, also known as CK1alpha or CK1a). CSNK1A1 regulates the Wnt/β-catenin signaling pathway and has dual functions in cell cycle progression and cell division (Knippschild et al. 2005; Schittek and Sinnberg 2014; Cai et al. 2018). CSNK1A1 is known to influence tumor progression; however, its role as a tumor suppressor or oncogene is tumor type–dependent (Järås et al. 2014; Schittek and Sinnberg 2014; Lantermann et al. 2015; Cai et al. 2018), and CSNK1A1 has no known roles in PDAC. *CSNK1A1* has very low gene expression in normal pancreas but is overexpressed in PDAC (Jiang et al. 2018). We found that *CSNK1A1* shows significantly higher expression in the PDAC epithelium as compared to precursor lesions—premalignant pancreatic intraepithelial neoplasia (PanIN) (Fig. 7A) and intraductal papillary mucinous neoplasia (IPMN). We found no significant difference in *CSNK1A1* expression in the stroma between PDAC and precursor lesions. 3′ RACE showed that *CSNK1A1* has both the short and long 3′-UTR forms in PDAC cells as predicted by our computational analysis (Supplemental Fig. S5A).

To provide genetic evidence for the role of CSNK1A1 in PDAC cell growth, we knocked down *CSNK1A1* in Suit2 and MIA PaCa-2 cells with three short hairpin RNAs (shRNA) (Fig. 7B; Supplemental Fig. S5B). *CSNK1A1* knockdown decreased both cell proliferation and clonogenic growth of PDAC cells (Fig. 7C–E; Supplemental Fig. S5C,D), with Suit2 cells showing increased sensitivity to *CSNK1A1* loss. The strongest phenotypic effects were associated with the most efficient knockdown (shRNA 3) in both cell lines. We then investigated the potential of CSNK1A1 as a pharmacological target in PDAC biology with the widely used small molecule inhibitor D4476 (Rena et al. 2004; Lantermann et al. 2015; Jiang et al. 2018). In concordance with the genetic evidence, although MIA PaCa-2 and Suit2 cells were both sensitive to D4476 treatment, Suit2 cells displayed a 10-fold lower IC50 (Fig. 7F). Both cell lines also showed dose-dependent decreases in cell proliferation (Fig. 7G; Supplemental Fig. S5E) and clonogenic growth in the presence of the inhibitor (Fig. 7H,I; Supplemental Fig. S5F). Therefore, we identify CSNK1A1 as a putative drug target in PDAC and reveal the potential of cancer-specific APA analyses to identify mechanisms of altered gene expression driving cancer pathogenesis.

## Discussion

Dysregulated gene expression is a cardinal feature of cancer (Yaffe 2019). However, how gene expression is altered in cancer and whether the processes driving this dysregulation can be targeted therapeutically are areas of active investigation. APA has recently been identified as a candidate driver of gene expression dysregulation. APA factors frequently show aberrant expression in cancer, modulate the expression of known oncogenes and tumor suppressors, and knockdown studies have highlighted their relevance to the cancer phenotype (Masamha et al. 2014; Miles et al. 2016; Chen et al. 2018b; Mitra et al. 2018; Tan et al. 2018; Chu et al. 2019). Whole-genome CRISPR and shRNA screens have also revealed the requirement for several APA factors in pancreatic cancer cell growth (https://www.depmap.org). Global analyses have revealed widespread 3′-UTR changes across multiple cancer types, uncovering recurrent alterations common across the cancer spectrum (Xia et al. 2014; Grassi et al. 2016; Feng et al. 2017; Ye et al. 2018). Recent findings suggest that although some APA events are widely shared across cancers, many are tumor type–specific (Xue et al. 2018). Despite this observation, there have been few attempts to study APA in a single tumor type with sufficient power to identify tumor-specific alterations and vulnerabilities.

To our knowledge, this study represents the first global, in-depth, single-cancer view of APA, and the first examination of APA in PDAC clinical samples. The only previous study of APA in PDAC showed gemcitabine-induced 3′-UTR shortening of the transcription factor ZEB1 in the context of drug resistance (Passacantilli et al. 2017). Previous APA analyses combined multiple tumor types and used tumor-adjacent tissue as a “normal” control. However, matched tumor-adjacent normal tissues are known to represent a state that significantly differs from healthy, normal tissues and may therefore miss critical APA events (Aran et al. 2017). Furthermore, there are insufficient numbers of tumor-adjacent pancreatic samples within TCGA for a statistically stringent analysis. Therefore, we attempted to address these issues by using normal pancreas RNA-seq information from the GTEx project. A limitation of comparing independently collected data sets is the inherent disparity between them (Lappalainen et al. 2013). We attempted to rectify this by (1) confirming that the two data sets underwent identical library preparation methods on the same type of sequencing platform; (2) following identical data processing pipelines from the raw sequencing data to generate the coverage data; and (3) validating our top hits in an independent microdissected data set. Consistent with previous publications comparing TCGA and GTEx data sets, we compared the expression of housekeeping genes between the two data sets and observed minimal differences. Because batch effects cannot be completely ruled out, we performed experimental validation of several candidate APA-regulated genes, including *PAF1* and *ALDOA*, highlighting the robustness of our approach and relevance of our findings to PDAC biology. Furthermore, this approach will allow the analysis of APA in other tumor types for which little tumor-adjacent material is present in TCGA.

Multiple insights from our analyses are noteworthy. We find that APA events are recurrent and widespread across PDAC patients. For example, 68 genes were shortened and 28 genes were lengthened in >90% of the patient cohort. This supports the conjecture that APA dysregulation is a frequent event in PDAC. In support of this hypothesis, we find that several APA factors are highly expressed in PDAC, including *CSTF2* (Supplemental Fig. S6). CSTF2 has previously been implicated as a promoter of lung and bladder cancer, through the regulation of *ERBB2* and *RAC1* 3′ UTRs, respectively (Chen et al. 2018b; Mitra et al. 2018). We find frequent 3′-UTR alterations in several PDAC-relevant genes whose mechanisms of regulation were previously unknown, including *PAF1*, *ALDOA*, and *FLNA*. Many of the shortened 3′ UTRs showed increased gene expression, providing the first collection of 3′-UTR alterations that correlate with gene expression changes in PDAC. We were able to functionally validate these through luciferase reporter assays, highlighting the robustness of our analysis. Consistent with pan-cancer APA analyses, we find enrichment for pathways such as smooth muscle contraction and mRNA 3′-end processing (Lianoglou et al. 2013; Xia et al. 2014; Chen et al. 2018a). However, we also find enrichment for pathways and processes implicated in PDAC biology, including protein metabolism, receptor tyrosine kinase signaling, and signaling by RHO GTPases. Indeed, the link between 3′-UTR alterations and cancer metabolism has been identified in previous pan-cancer APA analyses (Xia et al. 2014). We also find an unexpected enrichment for loss of binding sites for tumor-suppressive miRNAs in frequently lost 3′-UTR regions. Therefore, we propose that APA is an underappreciated mechanism of gene dysregulation in PDAC, driving the expression of growth-promoting genes through disruption of miRNA-mediated regulation.

The extent of heterogeneity in proximal PAS usage across cancer patients has been largely overlooked in previous pan-cancer APA analyses. We found little heterogeneity in the extent of 3′-UTR proximal site usage in most genes (including housekeeping genes) in both normal and PDAC samples, again providing evidence for minimal batch effects. However, PDAC patients showed substantial heterogeneity in the extent to which their metabolic genes used the proximal PAS. This metabolic plasticity in turn could serve as a mechanism to deal with the fluctuating metabolic demands of cancer cells. These data support the possibility that APA may drive deregulation of cancer metabolism and tumor evolution by allowing for PAS choice plasticity of critical metabolic genes in PDAC.

Several studies have shown the power of APA analysis to improve expression-based prognostic markers. We report the first subset of 3′-UTR alterations that act as an independent prognostic indicator of PDAC outcome. Although several of the genes in this set are known regulators of tumorigenesis, including *SAT1*, many have not been implicated in PDAC biology and may represent new mediators of cancer phenotypes. The lost miRNA sites are enriched for tumor-suppressive miRNA families. In particular, we observed that patients who retain binding sites for a subset of five miRNAs survive longer than patients who lose them. This uncovers the prognostic role for a novel subset of miRNA mediators in PDAC.

Our in-depth analysis of APA in PDAC revealed a critical role for the druggable target CSNK1A1 in PDAC cell growth and survival. Although CSNK1A1 regulates Wnt signaling and the *TP53* pathway, mediators of PDAC progression, the relevance of CSNK1A1 to PDAC was previously unknown (Järås et al. 2014; Lantermann et al. 2015; Cai et al. 2018; Jiang et al. 2018). Furthermore, the mechanisms of regulation of CSNK1A1 in cancer are not well understood, although promoter methylation in melanoma has been reported (Sinnberg et al. 2010). Two *CSNK1A1* isoforms have been detected in HeLa cells, with the shorter isoform being generated from the use of an alternative PAS (Yong et al. 2000). We show that *CSNK1A1* exhibits increased expression in PDAC samples as compared to precursors, and that pharmacological and genetic blockade of CSNK1A1 attenuates PDAC cell proliferation and clonogenic growth. Therefore, our single-cancer approach can identify APA-regulated, disease-specific vulnerabilities.

Our computational analysis and experimental validation have revealed unexpected mediators of PDAC biology and broadened our understanding of the regulatory role of 3′-UTR sequence space in cancer. This comprehensive analysis reveals the scope of previously uncharacterized APA events in regulating functionally relevant PDAC genes, improving patient prognosis and driving tumor evolution. We propose that the landscape of 3′-UTR alterations in PDAC represents a novel avenue to better understand PDAC progression and identify new drug targets.

## Methods

### Data collection and preprocessing

Our study focused on PDAC tumors consistent with the histology of PDAC (*n* = 148). All raw RNA-seq data used in this study were downloaded via NCBI dbGaP after our request for controlled-access data was processed. This included 184 normal pancreas SRA files from GTEx (dbGaP accession phs000424.v8.p2) and 148 BAM files within the TCGA-PAAD cohort (https://portal.gdc.cancer.gov/). GTEx SRA files were aligned exactly according to the TCGA RNA-seq alignment pipeline using GENCODE v22 annotations. The bedGraph files were generated using BEDTools v2.26 (Quinlan and Hall 2010) and were supplied as input to the DaPars algorithm.

### DaPars analysis

The bedGraph coverage files were processed using DaPars (Python 2.7.13) for de novo identification of differences in 3′ UTR between tumor and normal samples. The output of DaPars is a matrix of PDUI scores for every sample (columns) for any given gene (rows). Each cell represented a PDUI score for a gene corresponding to the tumor or normal sample. The PDUI score for a given gene represents the percentage of distal PAS usage in the sample. A higher PDUI score represents greater usage of the distal PAS site. In order to compare 3′-UTR changes for a given gene between tumor and normal samples, the PDUI scores for the gene was averaged over tumor samples (MeanPDUI_T_) and normal (MeanPDUI_N_) samples. A change in the mean PDUI score between tumor and normal samples for each gene (ΔPDUI = MeanPDUI_T_ − MeanPDUI_N_) was computed as a measure of 3′-UTR differences. The significance of this difference was assessed by the algorithm using Fisher's exact test, which was further adjusted using the Benjamini–Hochberg (BH) procedure (Benjamini and Hochberg 1995) to control the false discovery rate using 0.05 as a threshold to select significant hits. The final output of our analysis after eliminating redundant transcripts (based on lowest FDR) and selecting for protein-coding transcripts was a matrix containing 2573 unique genes as rows and tumor/normal sample in each column (total 148 tumor + 184 normal = 332 columns). Given that our samples are not matched, we did not generate a ΔPDUI value for a given gene for every sample, rather we have a unique ΔPDUI value for every outputted gene. A similar analysis was performed with a subset of 69 high purity PDAC tumor samples (The Cancer Genome Atlas Research Network 2017).

### Bioinformatics analyses and statistical methods

#### Analysis of heterogeneity

The variance in PDUI scores across tumor samples (Var[Tumor]) as well as normal samples (Var[Normal]) was computed for every gene as a measure of its heterogeneity in PAS site usage across samples. The difference in variance/heterogeneity between the two data sets (Var[Normal] − Var[Tumor]) was plotted (R version 3.6.0) (R Core Team 2014). The significance of the difference for each gene was assessed using an F test of variances.

#### Heatmap analysis

A heatmap representing the extent of 3′-UTR alterations across PDAC patients was generated (R version 3.6.0). Given that we do not have a ΔPDUI value for each patient, we computed an estimate of ΔPDUI associated with each patient for any given gene, using the following approach. For each significant gene hit (|ΔPDUI| > 0.1, row in heatmap), the mean GTEx PDUI score was subtracted from the PDUI score for each TCGA PDAC patient to obtain a measure of ΔPDUI_patient_ (change in 3′-UTR length for that gene for each patient). We implemented unsupervised hierarchical clustering using Ward's minimum variance method, to minimize the inter-cluster Euclidean distances. Rows were similarly clustered to yield subsets of genes undergoing a higher degree of 3′-UTR shortening (red) or lengthening (blue). Five distinct subgroups are presented to visualize the patterns of widespread 3′-UTR shortening among patients. The mutational status of commonly altered PDAC genes and PDAC subtype for each TCGA patient was highlighted (The Cancer Genome Atlas Research Network 2017).

#### Survival analysis

We first fit a complex clinical model including diagnosis age, stage, grade, residual tumor, sex, and race. Residuals from this model reflect signal not explained by the standard clinical subspace. PDUI scores associated with the residuals were then screened to identify a multivariate subspace to study. We selected 20 features (genes) to study based on *P*-value order. Using repeated random starts, we selected K = 3 *k*-means clustering based on the elbow heuristic to define three prognosis groups based on the within/between sum of squares criterion. The prognostic value of this classification is described by standard Kaplan–Meier plot and the log-rank test. We determined that 85% of random restarts led to statistically significant log-rank tests for K = 3 suggesting that significant additional prognostic information in the PDUI subspace above the clinical data was stable as opposed to stochastic clustering.

#### Percentage of lost miRNA sites

Highly conserved miRNA binding sites and their genomic positions were downloaded from TargetScanHuman 7.2. This list, along with DaPars prediction of genomic coordinates of lost 3′ UTRs was used to plot the number of genes that lose at least one highly conserved miRNA binding site.

#### miRNA families preferentially associate with lost sites

To determine miRNAs associated with sites enriched in lost 3′ UTRs, miRNA target predictions and the cumulative weighted context++ scores (CWCS) were downloaded from TargetScanHuman 7.2. CWCS estimates the predicted cumulative repression for a miRNA at the site. The lost miRNA binding sites in the shortened 3′ UTRs of PDAC patients were inferred from DaPars predictions. A weighted target site score was computed as the sum over all genes with shortened 3′ UTRs in tumor, with the CWCS of each target site for the miRNA multiplied by the normalized abundance of the gene's 3′-UTR form in which the predicted target site was present. The fold change (*f*) of the sum of weighted target site scores in lost 3′-UTR regions for PDAC tumor over normal was calculated (*f* = score in tumor/score in normal). The labels of the miRNA target sites were permuted to assess the significance of the fold change. One thousand such randomizations were performed and the mean (*m*) and standard deviation (*s*) of the fold changes across the randomized data sets was computed. The significance of the fold change was computed in form of the *Z*-score defined as (*f* − *m*)/*s*. A lower *Z*-score indicates that the loss in miRNA binding sites is higher than that expected by chance.

#### miRNA prognostic signature

We quantified the impact of APA-based loss of miRNA binding as follows:

$$X_{m,i}=\Sigma_{g}(1-PDUI_{i,g})\times A_{g,m},$$

where *A*_*g,m*_ is an indicator function that the short versus long 3′ UTR of the gene *g* contains the binding site for miRNA *m*, the impact to the *i*th person is *X*_*m,i*_. We used Sure Independence Screening (SIS) to search through all affected miRNAs and identify features that were associated with survival univariately (Fan and Lv 2008). To study the multivariate effect, we reorganized cases using the Euclidean distance between SIS selected features, visualized with t-SNE, and defined clusters with model-based Gaussian clustering using the BIC criterion to select cluster number. Survival differences were tested across all groups by the log-rank test and were visualized by Kaplan–Meier estimate. We performed the analysis of deviance test of the nested Cox regression model: clinical variables versus clinical variables + miRNA clusters which was statistically significant (*P* = 0.02) suggesting that addition of miRNA clusters improved the model. The pattern of loss of miRNA binding sites across patient clusters was visualized for a subset of miRNAs in a heatmap.

### Experimental methods

#### Cell lines and general reagents

MIA PaCa-2 and HEK293 cells were purchased from ATCC and cultured in DMEM media (Corning MT 10-013-CV) and 10% fetal bovine serum. Suit2 cells were obtained from Dr. David Tuveson (Cold Spring Harbor Laboratory). Cell lines were periodically verified to be mycoplasma free using the MycoAlert kit (Lonza LT07-701). All transfections were carried out using Lipofectamine 3000 (Thermo Fisher Scientific L3000008) per the manufacturer's protocol.

#### 3′ RACE assays

cDNA was generated from 1 µg RNA from MIA PaCa-2 as well as Suit2 cell lines using SuperScript II Reverse Transcriptase (Thermo Fisher Scientific 18064022) using the primer P: 5′-GACTCGAGTCGACATCGATTTTTTTTTTTTTTTTT-3′. To PCR amplify the 3′-UTR forms of candidate genes, a gene-specific forward primer spanning the stop codon of the gene was used in conjunction with a reverse primer P′: 5′-GACTCGAGTCGACATCG-3′ targeting the adapter region introduced by primer P. The PCR mixture was run on a 1.5% agarose gel and visualized using the ChemiDoc imaging system followed by analysis with Image Lab software (Version 6.0.0, Bio-Rad). An identical cDNA generation and PCR procedure was followed for RNA extracted from PDAC patient tumor samples. RNA from PDAC patient samples were obtained from Roswell Park Pathology Shared Resource. Approval of biospecimen use was granted by the Roswell Park IRB.

#### Luciferase reporter assays

MIA PaCa-2 cells were seeded at ∼10,000 cells per well in a 96-well white plate (Thermo Fisher Scientific 07-200-628). Three technical replicates were plated for each condition. The cells were transfected the next day at ∼60% confluency with 200 ng of *Renilla* luciferase reporter plasmid (pIS1 containing the 3′-UTR region of interest) (Supplemental Table S1) and 2 ng of firefly luciferase reporter control plasmid pIS0 per well. In cases in which the 3′-UTR lengths between the short and long form were significantly different, we ensured that equal molar amounts of the 3′-UTR constructs were transfected. Luciferase readings were measured 24 h post-transfection with the Dual luciferase reporter assay system (Promega E1910) using the Synergy H1 plate reader. The *Renilla* reporter reading was normalized to its corresponding firefly reading in every well to control for transfection efficiency.

#### Statistical analyses

All experimental findings presented were replicated in three or more independent experiments. Comparisons between two groups were performed using unpaired *t-*test with Welch's correction in GraphPad Prism 8. In general, *P* < 0.05 was considered significant, and the determined *P*-values are provided in the figure legends. Asterisks in graphs denote statistically significant differences as described in figure legends.

### Software availability

The code to preprocess RNA-seq data as well as the R code for all analyses is provided as Supplemental Code and is also available at GitHub (https://github.com/feiginlab/APA_PDA).

## Competing interest statement

The authors declare no competing interests.

## Supplementary Material

Supplemental Material
