# Supplementary material for: Alternative polyadenylation drives oncogenic gene expression in pancreatic ductal adenocarcinoma
Source: Genome Res. 2020 Mar;30(3):347–60. doi: 10.1101/gr.257550.119 (PMC7111527; doi:10.1101/gr.257550.119)
Supplement: Supplemental Material [file supp_30_3_347__index.html]

Alternative polyadenylation drives oncogenic gene expression in pancreatic ductal adenocarcinoma — Supplemental Material 

# Alternative polyadenylation drives oncogenic gene expression in pancreatic ductal adenocarcinoma

## Supplemental Material

- Supplemental\_Material.pdf
- Supplemental\_Code.zip
