# Supplementary material for: Alternative polyadenylation drives oncogenic gene expression in pancreatic ductal adenocarcinoma
Source: Genome Res. 2020 Mar;30(3):347–60. doi: 10.1101/gr.257550.119 (PMC7111527; doi:10.1101/gr.257550.119)
Supplement: Supplemental Material [file supp_gr.257550.119_Supplemental_Material.pdf]

Supplemental Material

Supplemental text

Details of methods and associated references not included in the main text are provided below.

Bioinformatics analyses and statistical methods

Permutation test to identify ΔPDUI thresholds. To determine an appropriate ΔPDUI threshold to identify shortened/lengthened genes, we performed a permutation test (n=10000) by rearranging the tumor and normal labels on the PDUI scores for all genes. For each gene, we computed the value of ΔPDUI= $MeanPDUI_T$ - $MeanPDUI_N$ in every permutation. This yielded a distribution of ΔPDUI values for each gene. We computed the p-value for the thresholds of ±0.15, ±0.1, ±0.05 for every gene distribution (i.e. fraction of observed ΔPDUI scores > x and ΔPDUI scores< -x, x=threshold). These p-values were adjusted for multiple comparisons via the Holm correction method (p_{adj}). For the threshold of ±0.05, 23.8% of the genes showed a p_{adj} >0.05, suggesting that this threshold can lead to multiple false positives. However, 0 genes showed p_{adj} >0.05 for the ±0.1 and ±0.15 thresholds. This suggests that ±0.1 is a stringent threshold to identify shortened/lengthened genes with minimum false positives/negatives.

Pathway analysis. PANTHER (Protein ANalysis Through Evolutionary Relationships) was used for pathway analysis (Mi et al. 2019; Mi and Thomas 2009). The statistical overrepresentation test was used to statistically determine over or under-representation of reactome pathways in comparison to the reference list (all human genes in the PANTHER database) using Fisher's exact test (FDR <0.05).

Differential gene expression analysis. Differential gene expression analysis between TCGA-PAAD and GTEx normal pancreas samples was performed using DESeq2 (Love et al. 2014). DESeq2 normalized counts was used to compare expression levels of housekeeping genes (Eisenberg and Levanon 2003) between the tumor and normal datasets. For differential gene expression between the two datasets, genes showing (1) Fold change > 1.5 (2) FDR<0.05 were considered differentially expressed. The association between PDUI score and gene expression was plotted in R version 3.6.0. Fisher's exact test was performed to test for any significant association between 3' UTR shortened and upregulated genes.

Antibodies and general reagents.

All primers used in this study were purchased from Integrated DNA Technologies (IDT) and PCR reactions were performed using Q5 Hot start DNA polymerase (Cat# M0493L, NEB).

cDNA synthesis was carried out using SuperScript II Reverse Transcriptase (Cat# 18064022, Thermo Fisher Scientific). The *PAF1* reverse complement control construct, miRNA site mutations in *ALDOA* 3' UTR as well as mutations at the proximal PAS of long 3' UTRs were introduced using NEB Builder HiFi DNA assembly (Cat# E2621S, NEB). The *Renilla* reporter plasmid pIS1 (Plasmid# 12179) as well as the firefly plasmid pIS0 (Plasmid# 12178) were purchased from Addgene. Luciferase assays were performed using Dual Luciferase Reporter Assay System (Cat# E1910, Promega). For *CSNK1A1* drug studies, the small molecule inhibitor D4476 (Cat# 13305, Cayman Chemical) was dissolved in DMSO (Cat# S1078, Selleckchem) at a stock concentration of 20mM. For dose-response measurements and certain cell proliferation experiments, cell viability was assessed using CellTiter-Glo (Cat# G7571, Promega). 3 distinct predesigned shRNAs (sh1:Cat# V2LHS_176052, sh2:Cat# V2LHS_221905, sh3:Cat# V2LHS_263361) against *CSNK1A1* were procured from a commercial shRNA library (Dharmacon) from the Roswell Park Gene Modulation core. Primary antibodies used in this study included a polyclonal antibody against CSNK1A1 (Cat# A301-991A-M, Bethyl labs) and a monoclonal antibody against actin beta (Cat# 3700S, Cell Signaling Technology). Secondary antibodies included horseradish peroxidase-conjugated goat anti-mouse (Cat# A4416, Sigma-Aldrich) and goat anti-rabbit (Cat# 45-000-682, Fisher Scientific) antibodies.

Cell lysis and RNA extraction.

MiaPaCa2 and Suit2 cells were grown to 100% confluence in 10cm plates. The cells were washed with 10mL PBS, and 1mL TRIzol was added to the cell culture plate. Cells were scraped, then incubated in a 1.5mL microcentrifuge tube for 5 minutes. 0.2mL of chloroform was added, mixed well and the tubes were incubated at room temperature for 2-3 minutes. The samples were centrifuged at 12000×g for 15 minutes at 4°C and the upper aqueous phase was transferred to a fresh tube. After addition and incubation with 0.5mL of isopropanol for 10 minutes, the samples were again centrifuged for 10 minutes. The supernatant was removed and the RNA pellet was washed with 75% ethanol. The pellet was dissolved in RNase-free water and the quality of RNA was assessed using a NanoDrop spectrophotometer.

Constructs for reporter assays.

The long and short 3' UTRs were PCR amplified from genomic DNA or BAC DNAs procured from RPCI-11 human BAC library resource at Roswell Park and subcloned into the *Renilla* luciferase vector pIS1 (Plasmid# 12179, Addgene) between the XbaI/EcoRV and NotI restriction sites. The primers were designed in accordance with 3' UTR length estimates obtained from the

3’ RACE and are outlined in Supplemental Table S1. The proximal PAS for all the long 3’ UTR constructs as well as the *PAF1* control reporter containing the intact short 3’ UTR followed by reverse complement of the long 3’ UTR segment was synthesized using HiFi assembly builder.

D4476 studies.

For dose-response measurements, MiaPaCa2 and Suit2 cells were seeded at a concentration of 2500 cells per well in a 96-well white plate. The next day, D4476 was titrated over a range of concentrations using the Tecan D300e Digital Dispenser and cell viability was measured 96h post drug titration using a CellTiter-Glo assay. For cell proliferation experiments, MiaPaCa2 or Suit2 cells were seeded at a concentration of 250 cells per well in a 96-well clear plate (Cat# 130188, Thermo Fisher Scientific). DMSO control or D4476 was dispensed at varying concentrations and imaged on the Cytation™ 5 Cell Imaging Multi-Mode Reader to image cell count (high contrast bright field) over time. For clonogenic experiments, MiaPaCa2 or Suit2 cells were seeded at a concentration of 250 cells per well and treated with different concentrations of D4476. The cells were allowed to grow over a period of 8-10 days after which they were fixed (10% methanol, 10% acetic acid) and stained with 0.5% crystal violet solution (in methanol). The plates were rinsed with PBS (137mM NaCl, 2.7mM KCl, 6.5mM Na2HPO4, 1.5mM KH2PO4), dried overnight and scanned. The resulting images were quantified using ImageJ (Version 1.50i). The images were uniformly thresholded and quantified for number of particles (colonies).

***CSNK1A1* knockdown experiments.**

Three different shRNAs against *CSNK1A1* gene as well as a non-targeting control shRNA (Con shRNA) were used to generate MiaPaCa2 or Suit2 control and CSNK1A1 knockdown cells. Knockdown was confirmed via immunoblotting. Briefly, samples were run alongside a molecular weight ladder (Cat# 26624, Thermo Fisher Scientific) on 10% SDS PAGE gels and then transferred to PVDF membranes (Cat# IPVH00010, Thermo Fisher Scientific) at 100V for 1 h. The membrane was blocked with 5% non-fat dry milk powder in PBST (PBS+ 0.1% Tween-20) for 1h and then incubated in the same buffer containing the primary antibody overnight on a shaker at 4°C. Polyclonal anti-CSNK1A1 (1:1000) and monoclonal actin beta (1:1000) were used to detect CSNK1A1 and actin beta respectively. The membrane was washed 4 × 5 min in PBS-T, followed by incubation with HRP-conjugated secondary antibodies (1:1000) for 1 h and then another 4 × 5 min washes. The blots were soaked with the ECL substrate (Cat# 32106, Thermo Fisher Scientific) and imaged. For cell proliferation experiments, control and CSNK1A1

knockdown Suit2 cell lines were seeded at a concentration of 250 cells per well in a 96-well white plate. Cell proliferation was measured on Day 1, 3, 5 and 7 using a CellTiter-Glo assay. The same procedure was repeated for MiaPaCa2 cells with a seed concentration of 500 cells per well. For clonogenic assays, MiaPaCa2 or Suit2 cells were seeded at a concentration of 500 cells per well in a 6-well clear plate. The cells were allowed to grow over a period of 8-10 days, fixed, stained and quantified as described previously.

References

Eisenberg E, Levanon EY. 2003. Human housekeeping genes are compact. *Trends Genet* **19**: 362–5.

Love MI, Huber W, Anders S. 2014. Moderated estimation of fold change and dispersion for RNA-seq data with DESeq2. *Genome Biol* **15**: 550.

Mi H, Muruganujan A, Ebert D, Huang X, Thomas PD. 2019. PANTHER version 14: more genomes, a new PANTHER GO-slim and improvements in enrichment analysis tools. *Nucleic Acids Res* **47**: D419–D426.

Mi H, Thomas P. 2009. PANTHER Pathway: An Ontology-Based Pathway Database Coupled with Data Analysis Tools. *Methods Mol Biol* **563**: 123–140.

Supplemental Figure S1

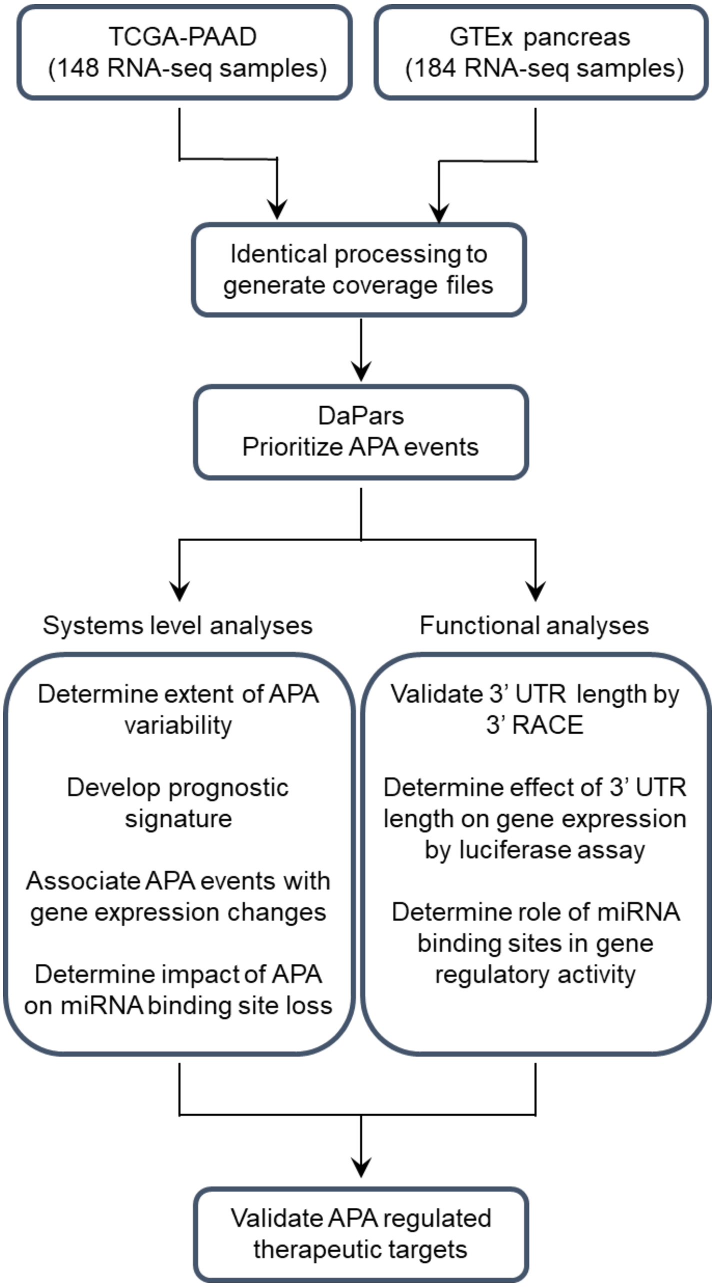

Supplemental Figure S1. Analysis flowchart. We identically processed raw RNA-seq data from the GTEx and TCGA-PAAD cohorts to analyze APA events in PDA. Predicted genes were further validated using a smaller high purity TCGA cohort and an independent micro-dissected dataset. The resulting genes were interrogated for associated APA trends, prognostic significance and gene expression changes.

Supplemental Figure S2

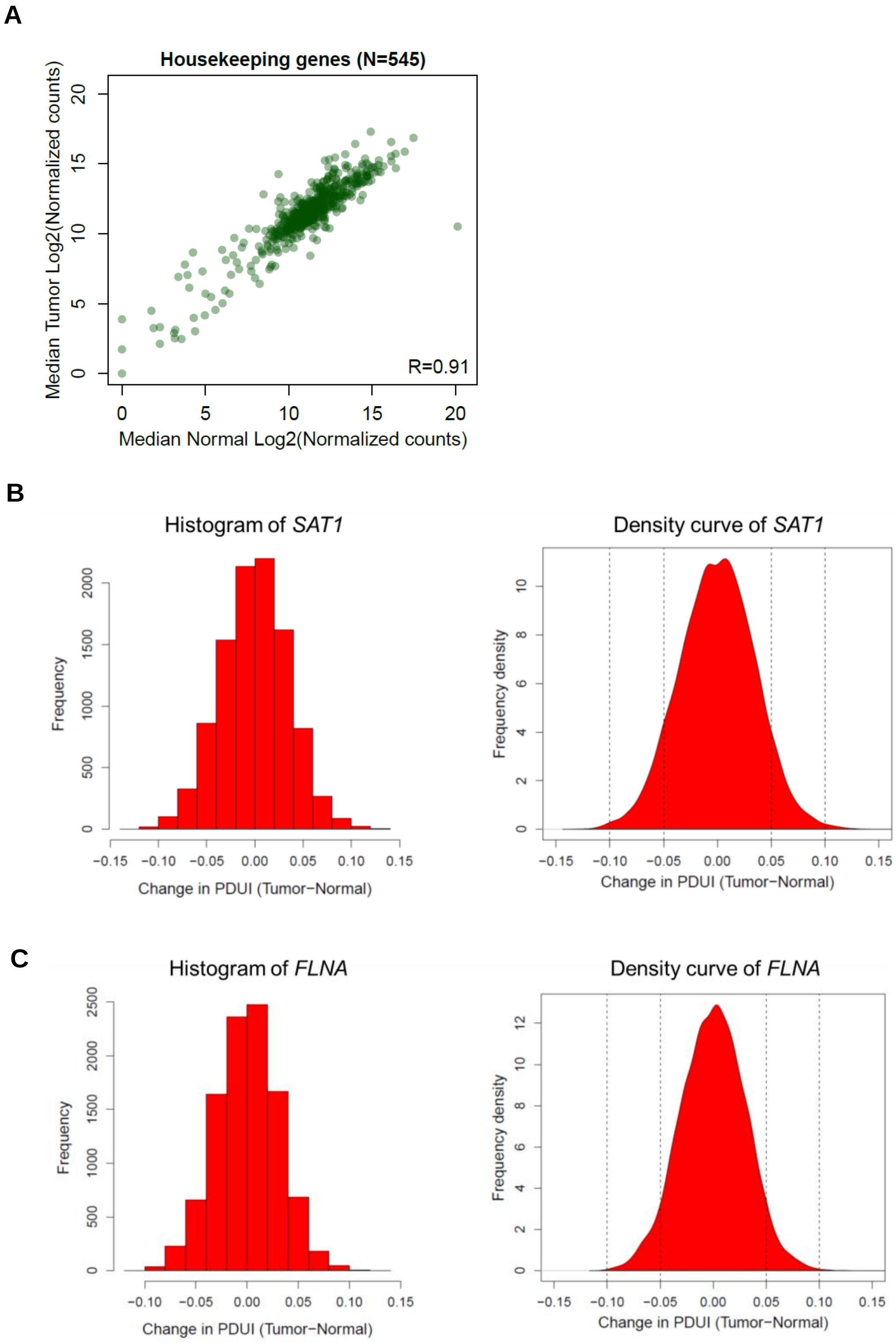

Supplemental Figure S2. Comparison of TCGA-PAAD tumors and GTEx normal pancreas. (A) Median log2 normalized counts of 545 housekeeping genes between TCGA-PAAD tumors and GTEx normal pancreas. Pearson correlation coefficient (R=0.91) is presented. (B) The distribution of ΔPDUI values of the top gene hit, *SAT1* over 10000 iterations of a permutation test is plotted as a histogram (left) and a density curve (right). (C) The same plot as in (B) for *FLNA*.

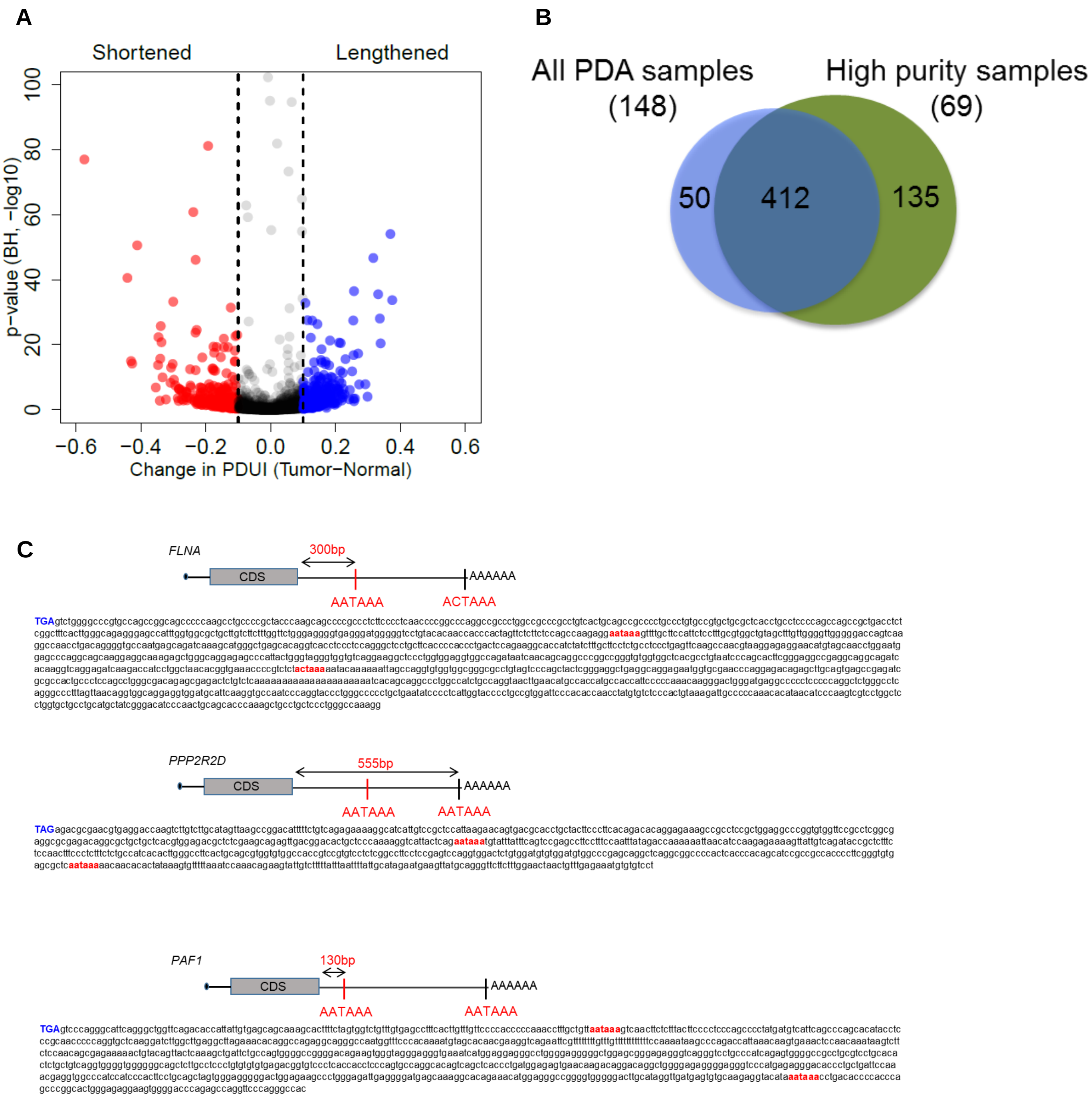

Supplemental Figure S3. Gene hits in the high purity TCGA-PAAD subset. (A) Volcano plot depicting significant gene hits (FDR<0.01) whose |DPDUI| > 0.1 in the 69 high purity samples (> 33% tumor content). (B) Venn diagram representing the overlap in significant gene hits between the DaPars analysis of 148 TCGA-PAAD samples and the 69 high purity TCGA-PAAD dataset. (C) 3’ UTR schematic and sequence of 3 example candidate genes (*FLNA, PPP2R2D* and *PAF1).* The stop codon is highlighted in blue and marks the beginning of the 3’ UTR sequence. The functional PAS sites estimated from 3’ RACE forms are highlighted in red.

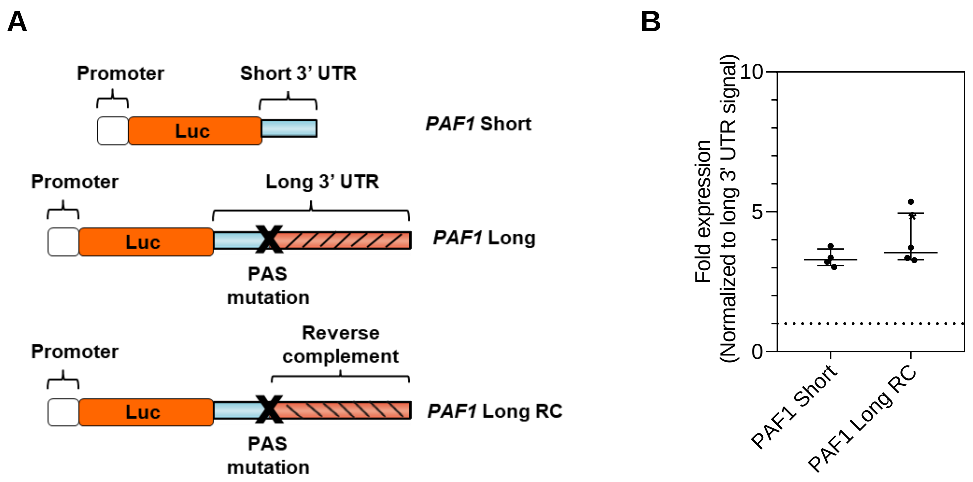

Supplemental Figure S4. Influence of PAF1 3' UTR length on protein expression. (A) Schematic illustrating the *PAF1* short, long and long RC (Reverse complement) reporter constructs. (B) Fold expression change of the luciferase reporter constructs *PAF1* short and *PAF1* long RC normalized to the *PAF1* long 3' UTR construct (n=4). The *PAF1* long 3' UTR expression is normalized to 1. Each whisker plot denotes the median as the center line and the minimum and maximum values as the whiskers.

Supplemental Figure S5

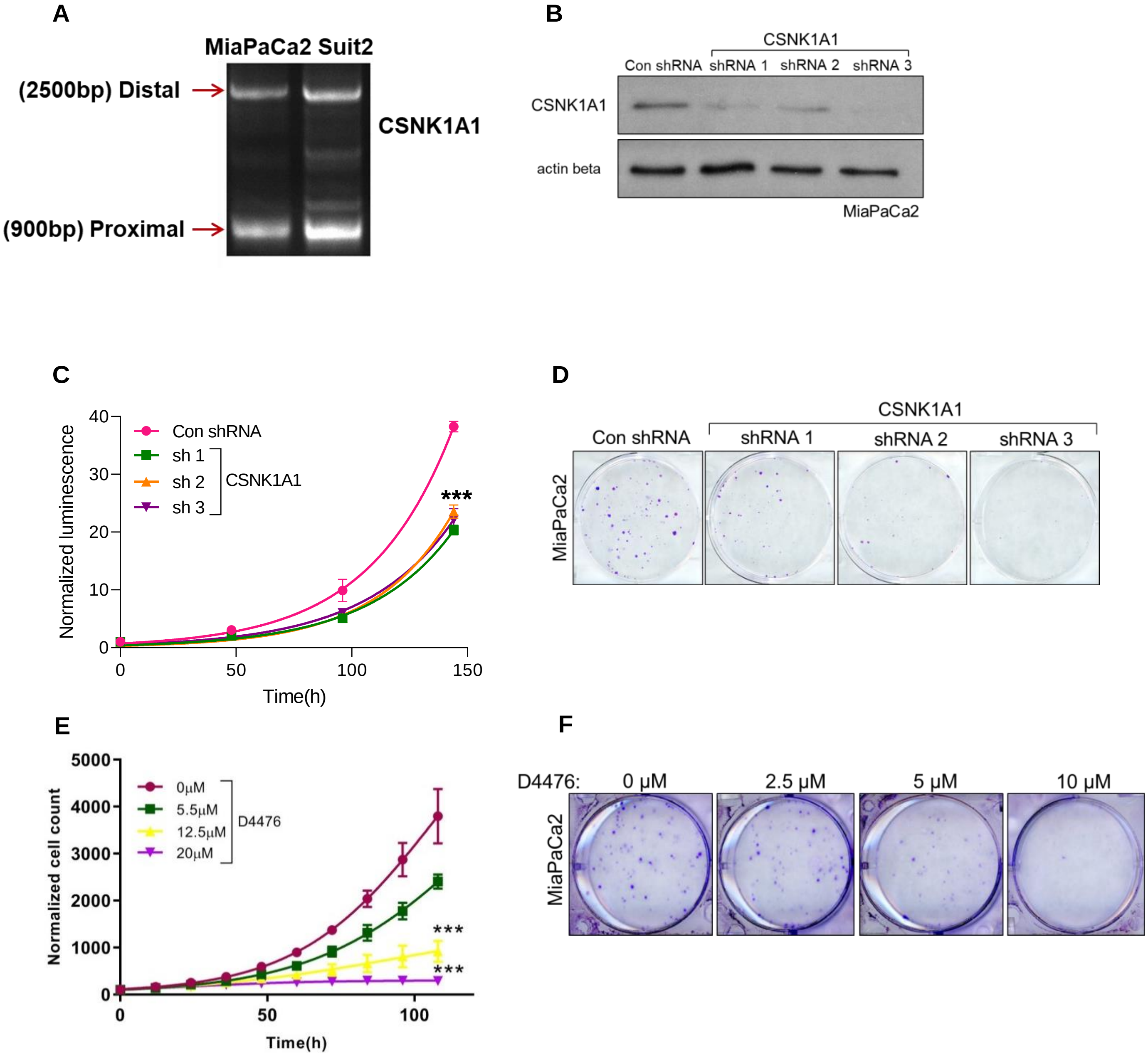

Supplemental Figure S5. CSNK1A1 is required for cell proliferation and is a putative drug target in PDA. (A) 3’ RACE of *CSNK1A1* in Suit2 and MiaPaCa2 cells (representative images from 3 independent experiments). (B) A representative blot (n=3) confirming CSNK1A1 knockdown in MiaPaCa2 cells with a non-targeting control shRNA (Con shRNA) or one of three different shRNAs targeting *CSNK1A1*. (C) Cell proliferation of MiaPaCa2 control and CSNK1A1 knockdown cells (n=3, ***p<0.005). (D) Clonogenic growth assay of MiaPaCa2 control and CSNK1A1 knockdown cells (n=3). (E) Cell proliferation of MiaPaCa2 cells treated with indicated doses of D4476 (n=3, ****p<0.001). (F) Clonogenic growth assay of MiaPaCa2 cells treated with indicated drug doses.

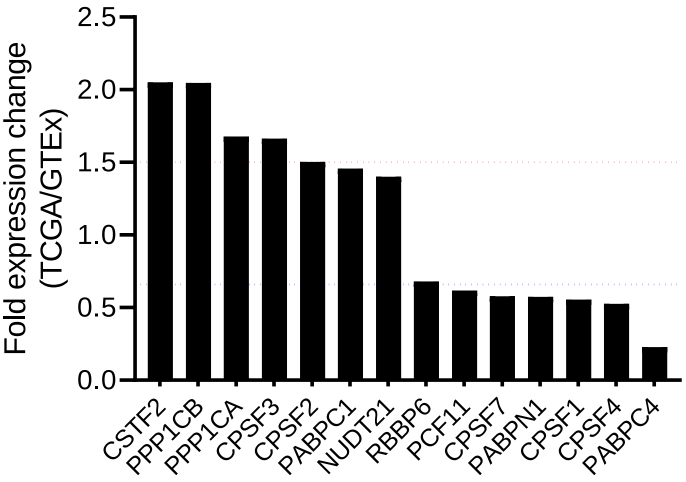

Supplemental Figure S6. APA factor expression in PDA. Fold expression change of core APA factors between TCGA-PAAD (tumor) and GTEx (normal pancreas). Dotted lines represent 1.5-fold (red) and 0.66-fold (blue) cutoffs.

Supplemental Table S1

Primers used to PCR amplify 3’ UTR short and long forms

PPP2R2DFwdXbaI	taagcaTCTAGAagacgcgaacgtgagga
PPP2R2DShortRevNot1	tgcttaGCGGCCGCcaataactttctcttggatgttaa
PPP2R2DLongRevNot1	tgcttaGCGGCCGCgaagaaccctgcataacttcattc
SAT1FwdXbaI	taagcaTCTAGAatatgctgcacttaagaatac
SAT1ShortRevNotI	tgcttaTCTAGAaaatgtgatttaacacaattac
SAT1LongRevNotI	tgcttaTCTAGActgaccaatcaacagggacc
ALDOAFwdXbaI	taagcaTCTAGAgcggaggtgttcccaggctgc
ALDOAShortRevNotI	tgcttaTCTAGAccacaagacacggacggccgac
ALDOALongRevNotI	tgcttaGCGGCCGCctgttaggtgaaggggcagagcc
TRIP10FwdXbaI	taagcaTCTAGAaccctgccagagacgggaag
TRIP10ShortRevNotI	tgcttaTCTAGAgaaacgtggtgttagatacttcc
TRIP10LongRevNotI	tgcttaTCTAGAcctgggcaactgggtgagac
PAF1EcoRVXbaI	taagcaGATATCCAGTGACTGAgtcccagggc
PAF1ShortRevNotI	tgcttaGCGGCCGCacctggggttgcgggaggt
PAF1LongRevNotI	TGCTTAGCGGCCGCgtggccctgggaacctggct
ENO1FwdEcoRV	taagcaGATATCGAAACCCCTTGGCCAAGTAA
ENO1ShortRevNotI	TGCTTAGCGGCCGCcctgaacactaaggacagacc
ENO1LongRevNotI	TGCTTAGCGGCCGCccttctggtctgaatatggc
RALGDSFwdXbaI	taagcaTCTAGAgggcatcctcccagggtc
RALGDS ShortRevNotI	tgcttaGCGGCCGCttgcccctccccaatcag
RALGDS LongRevNotI	tgcttaGCGGCCGCctggataaccctgcaagggtcc
FLNA FwdXbaI	taagcaTCTAGAgtctggggcccgtgcca
FLNA ShortRevNotI	tgcttaGCGGCCGCcccaacaaagctacagccacgc
FLNA LongRevNotI	tgcttaGCGGCCGCcctgcctcggcctcccgaa
