# Supplementary material for: Alternative polyadenylation drives oncogenic gene expression in pancreatic ductal adenocarcinoma
Source: Genome Res. 2020 Mar;30(3):347–60. doi: 10.1101/gr.257550.119 (PMC7111527; doi:10.1101/gr.257550.119)
Supplement: Supplemental Material [file supp_gr.257550.119_Supplemental_Code.zip › README.pdf]

All these bam files are controlled access data. We applied for permission to access TCGA controlled data via dbGAP and received it in about 3 weeks. GTeX pancreas samples were requested within the same project via dbGAP (dbGaP accession phs000424.v8.p2).

RNA seq Aligned reads of 178 pancreatic cancer patients were downloaded from TCGA (https://portal.gdc.cancer.gov/) using the following filters:

Projects>Pancreas>TCGA>Pancreatic Adenocarcinoma (diseasetype)>RawSequencing Data (data category)> RNASeq (Experimental Strategies).

TCGA-PAAD turns up with 185 cases. Click on the 185 link under cases. In the files tab select:

Aligned Reads (DataType)>RNA-Seq (Expermental Strategies)>BAM(DataFormat) (you are left with 182 cases)

Under the cases tab select:

Primary tumor, tumor and metastatic (Samples > Sample Type). We do not want normal samples and thus we are left with 178 cases. Histology of PDAC samples were recorded from supplementary information file in Raphael et al. 2017 and only PDAC samples were chosen (n=148). In order to download the data, add all the required files to cart. In order to download it on to a SGE cluster account, download the manifest file. Follow the instructions at https://docs.gdc.cancer.gov/Data_Transfer_Tool/Users_Guide/Data_Download_and_Upload/ in order to store it in file. All BAM aligned, PDA files were stored in the folder.

GTeX samples were in SRA format. The files were batch processed to FASTQ zipped format (SRAToFASTQ.sh). The FASTQ files were then assessed for quality via FASTQC . The summary report used to assess the quality of files. High quality files were then aligned exactly as per the TCGA RNA-seq pipeline (Code at: https://docs.gdc.cancer.gov/Data/Bioinformatics_Pipelines Expression_mRNA_Pipeline/) to yield BAM files.

Details of all GTEx and TCGA sample files used in this study is provided as csv files in the supplemental folder.

BAM files were converted to bedGraph files and used as an input to the DaPars algorithm (BAMToBEDGRAPH.sh). These files were supplied as input to DaPars. Gencode V22 annotations were used to extract 3' UTR information using Extract_Anno.py provided in the DaPars code files. Links to the extract UTR code, configure file format, DaPars code and instructions to run DaPars can be found at

http://lilab.research.bcm.edu/dldcc-web/lilab/zheng/DaPars_Documentation/html/DaPars.html.
